# The “Asthma-Polycystic Ovary Overlap Syndrome” and the Therapeutic Role of Myo-Inositol

**DOI:** 10.3390/ijms24086959

**Published:** 2023-04-09

**Authors:** Gabriella Guarnieri, Matteo Iervolino, Sara Cavallone, Vittorio Unfer, Andrea Vianello

**Affiliations:** 1 Department of Cardiac, Thoracic, Vascular Sciences and Public Health, University of Padova, 35128 Padova, Italy; 2 Systems Biology Group Laboratory, 00163 Rome, Italy; 3 The Experts Group on Inositol in Basic and Clinical Research (EGOI), 00161 Rome, Italy

**Keywords:** asthma, polycystic ovary syndrome, premenstrual asthma, inflammation, obesity, infertility, myo-inositol, α-lactalbumin

## Abstract

Asthma is a heterogeneous inflammatory disease characterized by abnormalities in immune response. Due to the inherent complexity of the disease and the presence of comorbidities, asthma control is often difficult to obtain. In asthmatic patients, an increased prevalence of irregular menstrual cycles, infertility, obesity, and insulin resistance has been reported. Given that these conditions are also common in patients with polycystic ovary syndrome (PCOS), we propose the definition of “asthma-PCOS overlap syndrome” to indicate a medical condition which shares characteristics of both diseases. The aim of this review is to analyze the links between asthma and PCOS and evaluate the therapeutic role of myo-inositol, a natural compound currently utilized in patients with PCOS, in the management of asthma patients.

## 1. Introduction

With an estimate of over 43 million new cases worldwide each year [1], asthma is one of the most common chronic diseases in the world. Globally, over 260 million people have poorly controlled asthma [2], making this condition the second leading cause of death among chronic respiratory diseases, with 450,000 deaths in 2017 [3]. Indeed, approximately 5% of asthmatics do not respond to standard therapy and are classified as “difficult to control”. Failure to achieve adequate control can be attributed to several factors, including comorbidities, which may impair the response to therapy and complicate patients’ clinical course. Pathophysiological mechanisms of asthma involve immune system cells (mast cells, eosinophils, neutrophils, and innate lymphoid cells), cytokines, and genetic factors [4].

In asthmatic patients, the most common symptoms include recurrent or episodic wheezing, shortness of breath, chest tightness, and cough, mainly occurring during night time or early in the morning, after exercise or as a result of exposure to allergens/cold air, or after intake of medications such as acetylsalicylic acid or β-blockers. Clinical suspicion of asthma is confirmed by the results of pulmonary function tests, which may detect a partially or fully reversible airflow limitation. Given that baseline pulmonary function evaluation may result in the normal range during intercritical periods, a more detailed assessment, including methacholine challenge test, serial peak flow monitoring, and measurement of airway inflammation may become essential to make a correct diagnosis [5,6].

Sputum eosinophil count is important for assessing asthma phenotype and predicting the response to biologic therapies. In the patient workup, it is also critical to evaluate risk factors such as allergic diathesis or occupational exposure. In-depth questionnaires may aid in the diagnostic path of bronchial asthma [7].

Most asthmatic patients have multiple comorbidities including rhinitis, sinusitis with or without nasal polyps, airway infections, gastro-esophageal reflux disease, obesity, sleep-related breathing disorders, anxiety and depression, and polycystic ovary syndrome (PCOS). Adequate treatment of comorbidities is essential to achieve satisfactory asthma control [8].

Gender differences should be taken into consideration when deciding therapeutic strategies in asthma patients [9,10,11,12]. Indeed, the risk of hospitalization for asthma exacerbation in adult women of childbearing age is higher than men, whereas levels are similar when considering the postmenopausal period [13,14].

A high prevalence of irregular menstrual cycles, infertility, obesity, and insulin resistance was reported in asthmatic women. These conditions are also present in patients with PCOS, a disorder which can be considered the most common reproductive endocrine disease [15,16], based on an estimated prevalence varying between 4% and 20%, [17,18,19,20,21]. Diagnostic criteria adopted for PCOS are outlined in Table 1.

PCOS is a complex condition characterized by metabolic abnormalities and increased expression of androgens leading to exacerbation of acne, hirsutism, and alopecia, which may impair women’s health status and quality of life [22]. PCOS is also associated with an increased risk of mood disturbances, in particular severe depression and anxiety [23]. Because patients with PCOS have a higher probability of developing asthma [24,25] and the number of prescriptions of asthma medications was found to be significantly higher among women with PCOS compared to the general female population [26], a link between asthma and PCOS was hypothesized [15,16].

**Table 1:** Diagnostic criteria for polycystic ovary syndrome.

The European Society of Human Reproduction and Embryology and the American Society for Reproductive Medicine criteria (2003) [25]. (2 out of 3 criteria must be fulfilled)	Oligo/anovulation (oligomenorrhea or amenorrhea)	Clinical and/or biochemical hyperandrogenism	Polycystic ovaries (12 or more follicles, with a diameter of 2–9 mm and/or 10 mm ovarian volume)
The Androgen Excess and Polycystic Ovary Syndrome Society criteria (2009) [26]. (All criteria must be fulfilled)	Hyperandrogenism	Ovarian dysfunction (oligo-anovulation and/or polycystic ovaries)	Exclusion of other androgen-related disorders

### Asthma Endotypes

In an effort to optimize the approach to the diagnosis and management of asthma patients, a classification of the disease based on different endotypes has been adopted in recent years. Endotypes can be distinguished according to the predominant inflammatory pathway and related biomarkers. They can be categorized as type 2-high (T2-high) endotypes, presenting with eosinophilic airway infiltrate and T_H_2-dependent cytokine overexpression (IL-4, IL-5, and IL-13) and type 2-low (T2-low) endotypes, showing neutrophilic and pauci-granulocytic airway infiltrates promoted by IL-6, IL-8, IL-17, IL-22, and epithelial cell-derived cytokines belonging to type 1 and 3 immunity [27]. Most patients with T2-high endotype report early disease onset, although a clear age-related cut-off has not yet been identified. In patients with T2-high asthma, biomarkers have been identified (i.e., sputum and blood eosinophils, exhaled nitric oxide, and blood IgE levels) that can be used to predict the response to biologic treatment [28].

Unlike this, T2-low endotype patients commonly report later disease onset and symptoms that are usually absent during childhood. Moreover, the prevalence of both female gender and obesity is unexpectedly high. Of importance, patients with T2-low asthma do not respond effectively to corticosteroid therapy [28,29].

**Figure 1:**
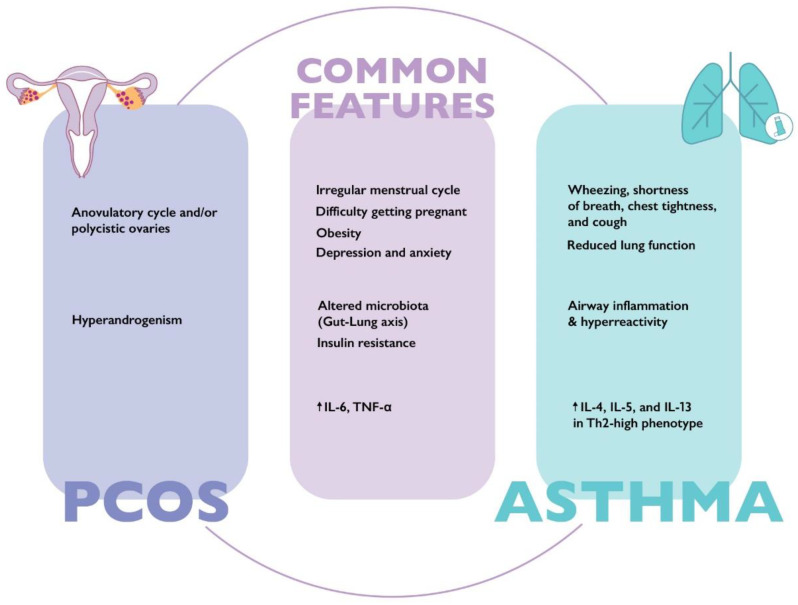
Main clinical and laboratory characteristics of asthma and polycystic ovary syndrome. IL-4: interleukin 4; IL-5: interleukin 5; IL-6: interleukin 6; IL-13: interleukin 13; TNF-α: tumor necrosis factor alpha; Th2: T helper 2.

Both asthma and PCOS are multifactorial, chronic, and very complex diseases. For this reason, identifying common pathways could allow us to optimize the therapeutic approach to both conditions. The aim of this review is to identify pathophysiological and clinical similarities between asthma and PCOS (Figure 1) and evaluate whether myo-inositol (myo-Ins), a treatment currently utilized in patients with PCOS, may also have benefits in subjects with asthma.

## 2. Clinical Overlap between Asthma and PCOS

An increasing number of studies has described common features between asthma and PCOS. Clinical similarities will be discussed below.

### 2.1. Obesity

Even though its role is not yet completely defined, obesity is one of the major factors influencing the severity and progression of asthma [30]. Given that it is often associated with poor response to medical treatment, obesity may influence the clinical course of asthma patients. Indeed, a higher dose of inhaled and oral asthma medications are commonly prescribed in obese compared to normal-weight asthmatic patients. Moreover, obese and overweight patients show a reduced response to inhaled corticosteroids (ICS) [31,32] and poor disease control [33]. Obese diabetic and prediabetic patients have an increased risk of developing asthma [34].

Obesity is associated with exacerbation of the inflammatory and immune response in asthma patients, depending on an increased synthesis of leptin, IL-6, and TNF-α. In addition, visceral fat accumulation may decrease lung volumes by reducing chest wall compliance [35].

Assad et al. reported that BMI can be considered a predictor of asthma onset in women, as it proved to be associated with, increased leptin serum concentration, and reduced adiponectin levels [36].

Visceral obesity, a common feature in patients with PCOS, may worsen hormone dysregulation and symptoms related to PCOS [37]. Moreover, women with PCOS were found to be at higher risk of obesity [38].

### 2.2. Insulin Resistance

Central obesity is associated with insulin resistance, which, in turn, may correlate with an increased risk of asthma-like symptoms, supporting the hypothesis that obesity and asthma may be linked through a common inflammatory pathway involving insulin resistance [39].

Obesity and insulin resistance may affect inflammatory pathways involving both the airway and the lung. Indeed, studies carried out in a mouse model showed that insulin sensitizers can be effective in the treatment of asthma. Of interest, the level of inflammatory mediators synthesized by alveolar epithelial cells can be normalized by insulin sensitizers [40,41,42]. Results of a study by Ma et al. showed that asthmatic mice receiving metformin had a significant reduction in both Th2 and non-Th2 inflammatory cytokines. Airway remodelling, goblet cell hyperplasia, collagen deposition, and airway smooth muscle hypertrophy were also reduced, due to restoration of 5′-adenosine monophosphate-activated protein kinase α (AMPKα) in the lungs [43].

Epidemiological studies showed that diabetic patients treated with insulin sensitizer (i.e., metformin) had lower risk of developing asthma and a reduced number of asthma-related hospitalizations [44,45].

Insulin sensitizer drugs alleviated airway hyperresponsiveness (AHR) in an obese, asthmatic animal model, suggesting that insulin resistance can play a critical role in bronchial reactivity. Unexpectedly, IL-6 and IL-1β levels were not changed after treatment [46,47].

Insulin resistance plays a key role in the pathophysiology of PCOS and was reported in up to 70% of patients with the disease [48,49]. Moreover, metformin was found to be effective in the management of patients with PCOS [50]. In addition to insulin resistance, uptake of glucose is defective in PCOS women, due to a reduction in GLUT-4, a glucose transporter. PCOS women also showed an alteration in Insulin Receptor Substrate 1 (IRS-1)-associated Inositol-triphosphate activity [51].

Polymorphisms of insulin receptor substrate 1 (IRS-1) have been found to increase fasting glucose levels and the risk of PCOS. In PCOS, insulin resistance is associated with genetic and epigenetic changes to signalling, as well as obesity [52].

The prevalence of metabolic syndrome is elevated in asthma patients [53]. Although the correlation between asthma and metabolic syndrome needs to be cleared, it may be influenced by various factors, including high BMI, increased resistin levels, dyslipidemia, and mitochondrial dysfunction [54,55,56].

### 2.3. Alterations in Gut Microbiota

Human microbiota includes archaea, protozoa, viruses, eukaryotes, and bacteria. Microbiota balances the innate and adaptive host defense, preserving homeostasis. It can interfere with vitamin absorption and is crucial for an effective immune response [57]. Stress, poor diet, and other conditions may affect the status of healthy microbiota [58].

Recent studies concluded that microbiota has a primary role in the pathophysiology of PCOS and that dysbiosis of gut microbiota should be considered as one of the possible causes of the disease. In particular, a diet rich in unsaturated fat and sugar can lead to increased gut permeability (‘‘leaky gut’’), resultinging in an increased passage of lipopolysaccharide (LPS) originating from Gram-negative colonic bacteria into the systemic circulation. The consequent activation of the immune system may interfere with insulin receptor function, impairing the insulin signaling pathway and causing insulin resistance. In turn, insulin resistance may increase the ovarian synthesis of androgens and affect follicle growth, leading to the onset of PCOS [59].

Alterations in microbiota can be also present in asthma patients [60,61]. Recent studies showed that gut and lung microbiota are closely interconnected (“gut–lung axis”) and can be involved in the pathogenesis of asthma [62]. In particular, a significantly reduced presence of Lactobacillus can be of great importance [57,60,61,62,63]. If gut microbiota is altered, endotoxins are generated that may contribute to chronic inflammation in asthma patients by activation of NF-κB pathway [64,65].

### 2.4. Menstrual Cycle Abnormalities

Premenstrual asthma (PMA) refers to the cyclical exacerbation of asthma symptoms immediately before or after menstrual cycle [11].

PMA is characterized by exacerbations which may occur during the periovulatory, preovulatory, or luteal phases, leading to an increased use of relief medication. The prevalence of PMA ranges between 11 and 45% of asthmatic patients and its presence correlates with an increased risk of hospitalization [12,66].

Respiratory symptoms (i.e., wheezing, shortness of breath, and cough attacks) may vary depending on the phase of menstrual cycle. Given that insulin levels also vary during the menstrual cycle, it has been hypothesized that insulin resistance may play a central role in asthma exacerbation [67]. Variations in insulin resistance in the different phases of the menstrual cycle are closely related to FSH level [68].

Calcaterra et al. suggested that estrogen may play a key role in the exacerbation of PMA symptoms. Estrogen receptors (ER) are present at alveolar level and may activate intracellular signaling cascades, including MAPK, ERK1/2, and phosphoinositide 3-kinase (PI3K) pathways. In particular, stimulation of ER-β receptors may increase inflammatory activity sustained by bronchial smooth muscle cells [12]. Of interest, administration of exogenous estradiol was found not to improve symptoms [69].

Whilst the role of estrogens and progesterone is still under debate, evidence suggests that androgens are important factors contributing to the association between PMA and PCOS [70]. A reduction in hormone levels during the luteal phase can lead to less severe exacerbation in patients with PCOS [71].

Menstrual irregularities are one of the major clinical manifestations that affect women with PCOS, including irregular, anovulatory or absent cycles [25]. As shown by Svanes et al., menstrual irregularities are significantly associated with asthma, particularly in women between the ages of 25 and 42 years. This association is less strong in menopausal women [72].

Real et al. reported that women with oligomenorrhea have reduced lung capacity and increased asthma prevalence. The authors hypothesized that insulin resistance may represent a common pathway [70]; however, conflicting results have been reported on the relationship between hormonal fluctuation and asthma exacerbation [11,73].

### 2.5. Infertility

PCOS is a major cause of infertility due to anovulatory cycles [74]. Asthma has been also associated with infertility [75]. Although the rates of childbirth are comparable between asthmatic and non-asthmatic women, Jöud et al. showed that the use of assisted reproductive technology (ART) was significantly more common among asthmatic women. Moreover, the rate of pregnancy loss was higher compared to non-asthmatic [76,77].

Although some researchers hypothesized that asthma medication use was correlated to infertility, no clear evidence has emerged in this regard. Alternatively, asthma per se could be a cause of infertility [78].

Vejen Hansen et al. demonstrated that the percentage of asthmatic women who seek fertility treatment was higher (12%) compared to non-asthmatic (8%). This difference was more evident in women older than 35 years (25% versus 13%). The authors suggested that pro-inflammatory cytokines involved in asthma onset could play a critical role in implantation of an embryo [79]. In particular, low levels of IL-6 were correlated with an increased chance of achieving pregnancy with in vitro fertilization (IVF) [80], whilst high levels were associated with an increased risk of miscarriage [81].

Taking into account the overlap between asthma and PCOS clinical features, we were prompted to propose the definition of “asthma-PCOS overlap syndrome” to indicate a medical condition which shares characteristics of both diseases. In our opinion, the potential role played by PCOS-associated imbalance of sex hormones and metabolic disorders in the proinflammatory pathways involved in the pathogenesis of asthma could support the view of a clinical entity integrating common molecular mechanisms. In this regard, previous Authors suggested that chronic systemic inflammation related to type 2 diabetes mellitus and/or other metabolic syndromes leading to increased levels of IL-6, TNF-α, and Pentraxin 3, and decreased synthesis of IL-10 and adiponectin, may represent the link between PCOS, airway inflammation, or even asthma [15].

**Figure 2:**
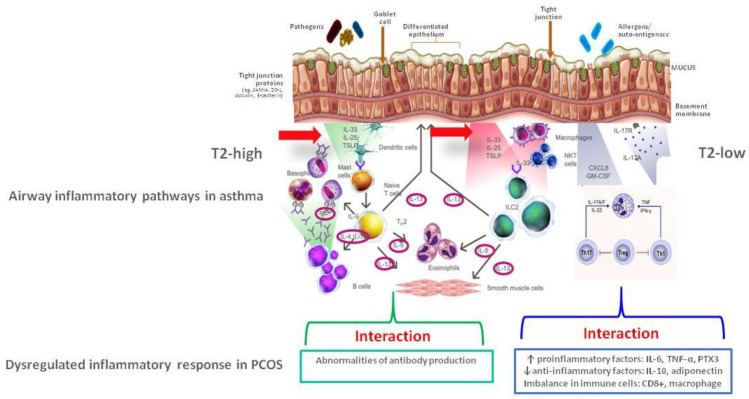
Potential interaction between molecular mechanisms behind polycystic ovary syndrome and airway inflammation in asthma. (PCOS = polycystic ovary syndrome; PTX3 = pentraxin 3).

The potential interaction between PCOS-related molecular mechanisms and airway inflammatory pathways in asthmatic patients is depicted in Figure 2.

## 3. The Therapeutic Role of Myo-Inositol in Patients with “Asthma-PCOS Overlap Syndrome”

### 3.1. Structural and Functional Characteristics of Myo-Inositol

A natural compound, myo-inositol (myo-Ins) raised considerable interest among clinicians, due to its safety profile and the growing evidence of its effectiveness in managing PCOS [82,83,84,85]. Myo-Ins is the most abundant inositol in the human body, showing different physiological functions. Indeed, it was found to be a G-protein coupled second messenger involved in hormone signaling, in particular insulin, follicle-stimulating hormone (FSH), thyroid stimulating hormone (TSH), serotonin, acetylcholine, and dopamine signaling [86,87,88,89,90].

As a second messenger of FSH, myo-Ins has been widely used for managing PCOS, in particular for facilitating ovulation. Moreover, myo-Ins can improve pregnancy outcomes in women undergoing ART [91,92,93], restore hormonal balance, reduce acne and hirsutism [94,95], and limit the probability of developing gestational diabetes (GDM) [96]. Importantly, it was found to be extremely safe, even at high doses [97]. Myo-Ins can increase insulin sensitivity and was proven to be as effective as metformin in improving metabolic control [98,99,100,101].

Myo-Ins is an essential component of lung surfactant, which in turn plays a fundamental role in reducing alveolar surface tension. The composition of lung surfactant includes surfactant protein A (SP-A) and D (SP-D), both of which act as a barrier to pathogens and modulate inflammatory response [102].

An alteration in surfactant synthesis was demonstrated in obese asthmatic patients, in particular a reduction in SP-A protein [103], which can be explained by increased secretory phospholipase A (2) [sPLA(2)] and eosinophil lysophospholipase activity [104]. It is important to note that myo-Ins is involved in the SP-A activation pathway via PI3K. Indeed, it can prevent SP-A-induced rise in macrophage mannose receptor (MR) expression through inhibition of PI3K function. PI3K plays a role in SP-A upregulation, and, in turn, SP-A induces a rise in IP3, activating the Ca^2+^/PLC/IP3 signal transduction pathway [105,106].

### 3.2. Therapeutic Role of Myo-Inositol in Lung Disorders

The efficacy and safety of myo-Ins in the treatment of lung diseases have been assessed by several trials. In a phase 1 study evaluating the safety, tolerability, and chemo-preventive effect of its oral administration, myo-Ins showed a protective effect on tracheo-bronchial mucosa, leading to a significant regression of bronchial dysplastic lesions. Moreover, it was proven to be safe even at high doses, causing mild side effects at gastrointestinal level [107].

In line with these results, myo-Ins showed a chemo-preventive effect on the development of bronchial dysplasia in smoking patients. A protective effect on lesion progression and the ability to reduce the serum level of inflammatory cytokines, in particular IL-6, were demonstrated [108]. Laganà et al. suggested that IL-6 reduction could be mediated by inositol-requiring enzyme 1 (IRE1), X-box-binding protein 1 (XBP1), and activation of transcription 3 (STAT3) pathways. Myo-Ins supplementation also increased surfactant concentration by stimulating PI3K [109]. A study conducted on premature infants with respiratory distress syndrome (RDS) showed that myo-Ins reduced the risk of lung damage and was associated with a lower mortality rate compared to the placebo group [110,111]. In this regard, a systematic review from the Cochrane library concluded that myo-Ins supplementation may significantly reduce short-term adverse neonatal outcomes and the incidence of bronchopulmonary dysplasia [112].

Several studies reported that myo-Ins has the potential to reduce inflammation and oxidative stress [113,114]. Of interest, myo-Ins was found to decrease the level of inflammation mediated by the NF-kB pathway in patients with severe asthma [114].

### 3.3. Routes of Drug Administration

When focusing on myo-Ins therapeutic effect on lung disease, inhalation route should be preferred. Indeed, inhalation route can be rapidly effective, reduce the risk of side effects, and avoid first-pass metabolism [115]. A pilot study demonstrated that nebulized myo-Ins was able to reduce symptoms and increase SpO2 levels in patients with various lung diseases, including asthma [116].

If orally administered, myo-Ins is absorbed at the intestinal level via sodium/myo-Ins transporters (SMIT1/2). A study by Orrù et al. found that a twice-dose 2 g oral myo-Ins in powder form could effectively cover 24 h. There are no sex differences in myo-Ins absorption [117]. Several factors can influence the absorption of myo-Ins, including the simultaneous intake of food or beverages containing sugar or sugar substitutes [118]. Coffee may also interfere with the absorption of myo-Ins [119].

Of interest, several studies showed that overall restoration of ovulation in PCOS women treated with myo-Ins was between 60% and 70% [120,121,122]. High body mass index (BMI), obesity, insulin resistance, and dysbiosis were identified as possible causes of “inositol resistance”.

To increase the proportion of responders to treatment, optimizing myo-Ins absorption is essential [122]. Alpha-lactalbumin(α-LA), a small protein with a molecular weight of 14.2 kDa, and the major whey protein of breast milk may contribute to creating a healthy gut environment. α-LA also has anti-inflammatory, muco-protective, and trophic properties, improving the absorption of micronutrients. For this reason, the hypothesis was advanced that it may increase oral absorption of inositol [123,124].

A study by Monastra et al. demonstrated that α-LA improved the absorption of myo-Ins both in vitro and in vivo as measured by plasma levels compared to myo-Ins alone. The study also evaluated the improvement of myo-Ins absorption in human intestine Caco-2 cells, showing a 4.5 times greater trans-epithelial passage of myo-Ins in the intestinal human cells if it was combined with 10 mg/mL of α-LA [125]. Montanino et al. conducted a clinical study to evaluate whether myo-Ins efficacy could be improved when combined with α-LA. Initially, myo-inositol was administered to anovulatory PCOS patients as monotherapy at a dose of 2 g twice a day for 3 months. As a result, ovulation was not restored in 38% of cases, who were considered “inositol resistant”. Subsequently, the “inositol resistant” subgroup was supplemented with 2 g of myo-Ins plus 50 mg α-LA twice a day for further 3 months, resulting in the restoration of ovulation in 86% of cases [126].

Different mechanisms have been hypothesized to explain why α-LA may improve myo-Ins absorption. First, α-LA can modify intestinal tight junctions by stimulating the secretion of glucagon-like peptide-2 (GLP 2) [127]. Intestinal tight junctions create a dynamic barrier between epithelial and endothelial cells and regulate the diffusion of molecules, especially nutrients and micronutrients. On the other hand, glucagon-like peptide-2, promotes the absorption of nutrients by stimulating intestinal cell proliferation and regeneration [128,129]. Modulating tight junction function and stimulating GLP 2, α-LA can increase the uptake of various natural compounds [128,130].

Second, due to its probiotic activity, α-LA promotes the growth of bacteria that may improve gut health, such as Lactobacilli and Bifidobacteria, which are commonly reduced in the microbiota of PCOS women. Furthermore, α-LA may adhere to intestinal cells and prevent infection by enteropathogenic bacteria such as *E. coli*, *S. typhiumurium* and *S. flexneri*, and *K. pneumonia* [131].

Third, α-LA exerts an anti-inflammatory activity through the inhibition of cyclooxygenase (COX) and phospholipase A2 enzymes. Indeed, studies on animal models showed a reduction in inflammatory cytokines IL-6 and Prostaglandin E2 (PGE2) [124].

A study conducted by D’Anna et al. in pregnant women with gestational diabetes (GDM) confirmed the efficacy of combining 2 g of myo-Ins with 50 mg of α-LA twice a day. In fact, after two months of combined treatment, a statistically significant reduction in insulin resistance and improved fetal growth was reported in the treatment group versus placebo. Moreover, a decrease in the number of women who required insulin treatment and pre-term birth was evident [132].

Finally, Hernandez-Marin et al. showed that the combined treatment of myo-Ins and α-LA improved hormonal and metabolic parameters in PCOS patients. In particular, HOMA-index, LH, and androstenedione improved after 3 and 6 months of treatment in both Italian and Mexican populations [133]. To date, randomized control trials aimed at demonstrating whether oral administration of myo-Ins combined with α-LA may be effective in patients with airway/lung disease, including asthma are lacking.

In the event of asthma flare-up, rapid relief of symptoms is a fundamental need for patients. For this reason, nebulized myo-Ins pharmacokinetic studies analyzing its action speed and duration are essential to evaluate its therapeutic role in exacerbated patients.

In summary, even though evidence on pharmacokinetics is limited and study results on its therapeutic effect are scarce, preliminary data on the possible use of myo-Ins in patients with asthma are encouraging.

## 4. Conclusions

Asthma is a heterogenous inflammatory disease characterized by abnormalities in the immune response. Categorizing asthma by phenotype and endotype may facilitate both diagnostic and therapeutic approach to the disease. Overlapping clinical features, in particular obesity and insulin resistance, suggest the possibility of common molecular mechanisms between non-Th2 asthma endotype and PCOS. We propose the definition of “asthma-PCOS overlap syndrome” to indicate a medical condition which shares characteristics of both diseases. Oral myo-Ins is effective in improving symptoms and quality of life in women with PCOS. Preliminary results support the hypothesis that administration of myo-Ins may be also beneficial in patients with asthma.

## Data Availability

Not applicable.

## References

[1] Mattiuzzi C., Lippi G. (2020). Worldwide asthma epidemiology: Insights from the Global Health Data Exchange database. Int. Forum. Allergy Rhinol..

[2] Song P., Adeloye D., Salim H., Dos Santos J.P., Campbell H., Sheikh A., Rudan I. (2022). Global, regional, and national prevalence of asthma in 2019: A systematic analysis and modelling study. J. Glob. Health.

[3] Cao Y., Chen S., Chen X., Zou W., Liu Z., Wu Y., Hu S. (2022). Global trends in the incidence and mortality of asthma from 1990 to 2019: An age-period-cohort analysis using the global burden of disease study 2019. Front. Public Health.

[4] Holgate S.T., Wenzel S., Postma D.S., Weiss S.T., Renz H., Sly P.D. (2015). Asthma. Nat. Rev. Dis. Prim..

[5] www.ginasthma.org.

[6] Khatri S.B., Iaccarino J.M., Barochia A., Soghier I., Akuthota P., Brady A., Covar R.A., Debley J.S., Diamant Z., Fitzpatrick A.M. (2021). American Thoracic Society Assembly on Allergy, Immunology, and Inflammation. Use of Fractional Exhaled Nitric Oxide to Guide the Treatment of Asthma: An Official American Thoracic Society Clinical Practice Guideline. Am. J. Respir. Crit. Care Med..

[7] Kaplan A.G., Balter M.S., Bell A.D., Kim H., McIvor R.A. (2009). Diagnosis of asthma in adults. CMAJ.

[8] Varkonyi-Sepp J., Freeman A., Ainsworth B., Kadalayil L.P., Haitchi H.M., Kurukulaaratchy R.J. (2022). Multimorbidity in Difficult Asthma: The Need for Personalised and Non-Pharmacological Approaches to Address a Difficult Breathing Syndrome. J. Pers. Med..

[9] Graziottin A., Serafini A. (2016). Perimenstrual asthma: From pathophysiology to treatment strategies. Multidiscip. Respir. Med..

[10] Calcaterra V., Nappi R.E., Farolfi A., Tiranini L., Rossi V., Regalbuto C., Zuccotti G. (2022). Perimenstrual Asthma in Adolescents: A Shared Condition in Pediatric and Gynecological Endocrinology. Children.

[11] Zhang G.Q., Ermis S.S.O., Rådinger M., Bossios A., Kankaanranta H., Nwaru B. (2022). Sex Disparities in Asthma Development and Clinical Outcomes: Implications for Treatment Strategies. J. Asthma Allergy.

[12] Jenkins C.R., Boulet L.P., Lavoie K.L., Raherison-Semjen C., Singh D. (2022). Personalized Treatment of Asthma: The Importance of Sex and Gender Differences. J. Allergy Clin. Immunol. Pract..

[13] Chowdhury N.U., Guntur V.P., Newcomb D.C., Wechsler M.E. (2021). Sex and gender in asthma. Eur. Respir. Rev..

[14] Senna G., Latorre M., Bugiani M., Caminati M., Heffler E., Morrone D., Paoletti G., Parronchi P., Puggioni F., Blasi F. (2021). Sex Differences in Severe Asthma: Results from Severe Asthma Network in Italy-SANI. Allergy Asthma. Immunol. Res..

[15] Xu Y., Zhou Z.Y., Pan J.X., Huang H.F. (2022). Associations Between Asthma and Polycystic Ovary Syndrome: Current Perspectives. Front. Endocrinol..

[16] Thuesen B.H., Husemoen L.L., Hersoug L.G., Pisinger C., Linneberg A. (2009). Insulin resistance as a predictor of incident asthma-like symptoms in adults. Clin. Exp. Allergy.

[17] Rasquin Leon L.I., Anastasopoulou C., Mayrin J.V. (2022). Polycystic Ovarian Disease.

[18] Gibson-Helm M., Teede H., Dunaif A., Dokras A. (2017). Delayed Diagnosis and a Lack of Information Associated with Dissatisfaction in Women With Polycystic Ovary Syndrome. J. Clin. Endocrinol. Metab..

[19] Deswal R., Narwal V., Dang A., Pundir C.S. (2020). The Prevalence of Polycystic Ovary Syndrome: A Brief Systematic Review. J. Hum. Reprod. Sci..

[20] Rotterdam ESHRE/ASRM-Sponsored PCOS Consensus Workshop Group (2004). Revised 2003 consensus on diagnostic criteria and long-term health risks related to polycystic ovary syndrome. Fertil. Steril..

[21] Azziz R., Carmina E., Dewailly D., Diamanti-Kandarakis E., Escobar-Morreale H.F., Futterweit W., Janssen O.E., Legro R.S., Norman R.J., Taylor A.E. (2009). Task Force on the Phenotype of the Polycystic Ovary Syndrome of The Androgen Excess and PCOS Society. The Androgen Excess and PCOS Society criteria for the polycystic ovary syndrome: The complete task force report. Fertil. Steril..

[22] Louwers Y.V., Laven J.S.E. (2020). Characteristics of polycystic ovary syndrome throughout life. Adv. Reprod. Health.

[23] Çetinkaya Altuntaş S., Çelik Ö., Özer Ü., Çolak S. (2022). Depression, anxiety, body image scores, and sexual dysfunction in patients with polycystic ovary syndrome according to phenotypes. Gynecol. Endocrinol..

[24] Htet T.D., Teede H.J., de Courten B., Loxton D., Real F.G., Moran L.J., Joham A.E. (2017). Asthma in reproductive-aged women with polycystic ovary syndrome and association with obesity. Eur. Respir. J..

[25] Hart R., Doherty D.A. (2015). The potential implications of a PCOS diagnosis on a woman’s long-term health using data linkage. J. Clin. Endocrinol. Metab..

[26] Glintborg D., Hass Rubin K., Nybo M., Abrahamsen B., Andersen M. (2015). Morbidity and medicine prescriptions in a nationwide Danish population of patients diagnosed with polycystic ovary syndrome. Eur. J. Endocrinol..

[27] Ricciardolo F.L.M., Sprio A.E., Baroso A., Gallo F., Riccardi E., Bertolini F., Carriero V., Arrigo E., Ciprandi G. (2021). Characterization of T2-Low and T2-High asthma phenotypes in real-life. Biomedicines.

[28] Wenzel S.E. (2012). Asthma phenotypes: The evolution from clinical to molecular approaches. Nat. Med..

[29] Logotheti M., Agioutantis P., Katsaounou P., Loutrari H. (2021). Microbiome Research and Multi-Omics Integration for Personalized Medicine in Asthma. J. Pers. Med..

[30] Tantisira K.G., Weiss S.T. (2001). Complex interactions in complex traits: Obesity and asthma. Thorax.

[31] Thompson C.A., Eslick S.R., Berthon B.S., Wood L.G. (2021). Asthma medication use in obese and healthy weight asthma: Systematic review/meta-analysis. Eur. Respir. J..

[32] Peters-Golden M., Swern A., Bird S.S., Hustad C.M., Grant E., Edelman J.M. (2006). Influence of body mass index on the response to asthma controller agents. Eur. Respir. J..

[33] Boulet L.P., Franssen E. (2007). Influence of obesity on response to fluticasone with or without salmeterol in moderate asthma. Respir. Med..

[34] Wu T.D., Brigham E.P., Keet C.A., Brown T.T., Hansel N.N., McCormack M.C. (2019). Association Between Prediabetes/Diabetes and Asthma Exacerbations in a Claims-Based Obese Asthma Cohort. J. Allergy. Clin. Immunol. Pract..

[35] Tiotiu A., Labor M., Nedeva D., Novakova S., Oguzulgen I.K., Mihaicuta S., Braido F. (2020). How to apply the personalized medicine in obesity-associated asthma?. Expert. Rev. Respir. Med..

[36] Assad N., Qualls C., Smith L.J., Arynchyn A., Thyagarajan B., Schuyler M., Jacobs D.R., Sood A. (2013). Body mass index is a stronger predictor than the metabolic syndrome for future asthma in women. The longitudinal CARDIA study. Am. J. Respir. Crit. Care Med..

[37] Glueck C.G., Goldenberg N. (2019). Characteristics of obesity in polycystic ovary syndrome: Etiology, treatment, and genetics. Metabolism.

[38] Naderpoor N., Shorakae S., Joham A., Boyle J., De Courten B., Teede H.J. (2015). Obesity and polycystic ovary syndrome. Minerva. Endocrinol..

[39] Carpaij O.A., van den Berge M. (2018). The asthma-obesity relationship: Underlying mechanisms and treatment implications. Curr. Opin Pulm. Med..

[40] Calixto M.C., Lintomen L., André D.M., Leiria L.O., Ferreira D., Lellis-Santos C., Anhê G.F., Bordin S., Landgraf R.G., Antunes E. (2013). Metformin attenuates the exacerbation of the allergic eosinophilic inflammation in high fat-diet-induced obesity in mice. PLoS ONE.

[41] Park C.S., Bang B.R., Kwon H.S., Moon K.A., Kim T.B., Lee K.Y., Moon H.B., Cho Y.S. (2012). Metformin reduces airway inflammation and remodeling via activation of AMP-activated protein kinase. Biochem. Pharm..

[42] Guo Y., Shi J., Wang Q., Hong L., Chen M., Liu S., Yuan X., Jiang S. (2021). Metformin alleviates allergic airway inflammation and increases Treg cells in obese asthma. J. Cell. Mol. Med..

[43] Ma W., Jin Q., Guo H., Han X., Xu L., Lu S., Wu C. (2022). Corrigendum: Metformin Ameliorates Inflammation and Airway Remodeling of Experimental Allergic Asthma in Mice by Restoring AMPKα Activity. Front. Pharm..

[44] Wu T.D., Fawzy A., Akenroye A., Keet C., Hansel N.N., McCormack M.C. (2021). Metformin Use and Risk of Asthma Exacerbation among Asthma Patients with Glycemic Dysfunction. J. Allergy Clin. Immunol. Pract..

[45] Wen L., Zhong W., Chai Y., Zhong Q., Gao J., Guan L., Zhang M., Huaiquan L., Haiyang Y., Qingxue W. (2020). Association of Metformin Use with Asthma Exacerbation in Patients with Concurrent Asthma and Diabetes: A Systematic Review and Meta-Analysis of Observational Studies. Can. Respir. J..

[46] Gu C., Loube J., Lee R., Bevans-Fonti S., Wu T.D., Barmine J.H., Jun J.C., McCormack M.C., Hansel N.N., Mitzner W. (2022). Metformin Alleviates Airway Hyperresponsiveness in a Mouse Model of Diet-Induced Obesity. Front. Physiol..

[47] Calco G.N., Maung J.N., Jacoby D.B., Fryer A.D., Nie Z. (2022). Insulin increases sensory nerve density and reflex bronchoconstriction in obese mice. JCI Insight..

[48] Barber T.M., Franks S. (2021). Obesity, and polycystic ovary syndrome. Clin. Endocrinol..

[49] Legro R.S., Castracane V.D., Kauffman R.P. (2004). Detecting insulin resistance in polycystic ovary syndrome: Purposes and pitfalls. Obs. Gynecol. Surv..

[50] Long C., Feng H., Duan W., Chen X., Zhao Y., Lan Y., Yue R. (2022). Prevalence of polycystic ovary syndrome in patients with type 2 diabetes: A systematic review and meta-analysis. Front. Endocrinol..

[51] Lashen H. (2010). Role of metformin in the management of polycystic ovary syndrome. Ther. Adv. Endocrinol. Metab..

[52] Zhao H., Zhang J., Cheng X., Nie X., He B. (2023). Insulin resistance in polycystic ovary syndrome across various tissues: An updated review of pathogenesis, evaluation, and treatment. J. Ovarian Res..

[53] de Boer G.M., Tramper-Stranders G.A., Houweling L., van Zelst C.M., Pouw N., Verhoeven G.T., Boxma-de Klerk B.M., In ’t Veen J.C.C.M., van Rossum E.F.C., Hendriks R.W. (2021). Adult but not childhood onset asthma is associated with the metabolic syndrome, independent from body mass index. Respir. Med..

[54] Karamzad N., Izadi N., Sanaie S., Ahmadian E., Eftekhari A., Sullman M.J.M., Safiri S. (2020). Asthma and metabolic syndrome: A comprehensive systematic review and meta-analysis of observational studies. J. Cardiovasc. Thorac. Res..

[55] Serafino-Agrusa L., Spatafora M., Scichilone N. (2015). Asthma and metabolic syndrome: Current knowledge and future perspectives. World J. Clin. Cases.

[56] Cardet J.C., Ash S., Kusa T., Camargo C.A., Israel E. (2016). Insulin resistance modifies the association between obesity and current asthma in adults. Eur. Respir. J..

[57] Huang J., Zhang J., Wang X., Jin Z., Zhang P., Su H., Sun X. (2022). Effect of Probiotics on Respiratory Tract Allergic Disease and Gut Microbiota. Front. Nutr..

[58] Conlon M.A., Bird A.R. (2014). The impact of diet and lifestyle on gut microbiota and human health. Nutrients.

[59] Tremellen K., Pearce K. (2012). Dysbiosis of Gut Microbiota (DOGMA)—a novel theory for the development of polycystic ovarian syndrome. Med. Hypotheses.

[60] Molyneaux P.L., Cox M.J., Wells A.U., Kim H.C., Ji W., Cookson W.O.C., Moffatt M.F., Kim D.S., Toby M.M. (2017). Changes in the respiratory microbiome during acute exacerbations of idiopathic pulmonary fibrosis. Respir. Res..

[61] Ramírez-Labrada A.G., Isla D., Artal A., Arias M., Rezusta A., Pardo J., Gálvez E.M. (2020). The influence of lung microbiota on lung carcinogenesis, immunity, and immunotherapy. Trends Cancer.

[62] Rastogi S., Mohanty S., Sharma S., Tripathi P. (2022). Possible role of gut microbes and host’s immune response in gut-lung homeostasis. Front. Immunol..

[63] Liu R., Zhang C., Shi Y., Zhang F., Li L., Wang X., Ling Y., Fu H., Dong W., Shen J. (2017). Dysbiosis of Gut Microbiota Associated with Clinical Parameters in Polycystic Ovary Syndrome. Front. Microbiol..

[64] Lad N., Murphy A.M., Parenti C., Nelson C.P., Williams N.C., Sharpe G.R., McTernan P.G. (2021). Asthma and obesity: Endotoxin another insult to add to injury?. Clin. Sci..

[65] Durack J., Lynch S.V., Nariya S., Bhakta N.R., Beigelman A., Castro M., Dyer A.M., Israel E., Kraft M., Martin R.J. (2017). Features of the bronchial bacterial microbiome associated with atopy, asthma, and responsiveness to inhaled corticosteroid treatment. J. Allergy Clin. Immunol..

[66] Sánchez-Ramos J.L., Pereira-Vega A.R., Alvarado-Gómez F., Maldonado-Pérez J.A., Svanes C., Gómez-Real F. (2017). Risk factors for premenstrual asthma: A systematic review and meta-analysis. Expert Rev. Respir. Med..

[67] Macsali F., Svanes C., Sothern R.B., Benediktsdottir B., Bjørge L., Dratva J., Franklin K.A., Holm M., Janson C., Johannessen A. (2013). Menstrual cycle and respiratory symptoms in a general Nordic-Baltic population. Am. J. Respir. Crit. Care Med..

[68] Yeung E.H., Zhang C., Mumford S.L., Ye A., Trevisan M., Chen L., Browne R.W., Wactawski-Wende J., Schisterman E.F. (2010). Longitudinal study of insulin resistance and sex hormones over the menstrual cycle: The BioCycle Study. J. Clin. Endocrinol. Metab..

[69] Ensom M.H., Chong E., Carter D. (1999). Premenstrual symptoms in women with premenstrual asthma. Pharmacotherapy.

[70] Becerra-Diaz M., Song M., Heller N. (2020). Androgen and Androgen Receptors as Regulators of Monocyte and Macrophage Biology in the Healthy and Diseased Lung. Front Immunol..

[71] Tan K.S., McFarlane L.C., Lipworth B.J. (1997). Modulation of airway reactivity and peak flow variability in asthmatics receiving the oral contraceptive pill. Am. J. Respir. Crit. Care Med..

[72] Svanes C., Real F.G., Gislason T., Jansson C., Jögi R., Norrman E., Nyström L., Torén K., Omenaas E. (2005). Association of asthma and hay fever with irregular menstruation. Thorax.

[73] Nejatbakhsh Samimi L., Fallahpour M., Khoshmirsafa M., Moosavi S.A.J., Bayati P., Baharlou R., Falak R. (2021). The impact of 17β-estradiol and progesterone therapy on peripheral blood mononuclear cells of asthmatic patients. Mol. Biol. Rep..

[74] Collée J., Mawet M., Tebache L., Nisolle M., Brichant G. (2021). Polycystic ovarian syndrome and infertility: Overview and insights of the putative treatments. Gynecol. Endocrinol..

[75] Gade E.J., Thomsen S.F., Lindenberg S., Backer V. (2014). Female asthma has a negative effect on fertility: What is the connection?. ISRN Allergy.

[76] Jöud A., Nilsson-Condori E., Schmidt L., Ziebe S., Vassard D., Mattsson K. (2022). Infertility, pregnancy loss and assisted reproduction in women with asthma: A population-based cohort study. Hum. Reprod..

[77] Bláfoss J., Hansen A.V., Malchau Lauesgaard S.S., Ali Z., Ulrik C.S. (2019). Female asthma and atopy-impact on fertility: A systematic review. J. Asthma Allergy.

[78] Gade E.J., Thomsen S.F., Lindenberg S., Backer V. (2016). Fertility outcomes in asthma: A clinical study of 245 women with unexplained infertility. Eur. Respir. J..

[79] Vejen Hansen A., Ali Z., Malchau S.S., Blafoss J., Pinborg A., Ulrik C.S. (2019). Fertility treatment among women with asthma: A case-control study of 3689 women with live births. Eur. Respir. J..

[80] Altun T., Jindal S., Greenseid K., Shu J., Pal L. (2011). Low follicular fluid IL-6 levels in IVF patients are associated with increased likelihood of clinical pregnancy. J. Assist. Reprod. Genet..

[81] Galazios G., Tsoulou S., Zografou C., Tripsianis G., Koutlaki N., Papazoglou D., Tsikouras P., Maltezos E., Liberis V. (2011). The role of cytokines IL-6 and IL-8 in the pathogenesis of spontaneous abortions. J. Matern. Fetal. Neonatal. Med..

[82] Unfer V., Nestler J.E., Kamenov Z.A., Prapas N., Facchinetti F. (2016). Effects of Inositol(s) in Women with PCOS: A Systematic Review of Randomized Controlled Trials. Int. J. Endocrinol..

[83] Kamenov Z., Gateva A. (2020). Inositols in PCOS. Molecules.

[84] Cantelmi T., Lambiase E., Unfer V.R., Gambioli R., Unfer V. (2021). Inositol treatment for psychological symptoms in Polycystic Ovary Syndrome women. Eur. Rev. Med. Pharm. Sci..

[85] Pkhaladze L., Russo M., Unfer V., Nordio M., Basciani S., Khomasuridze A. (2021). Treatment of lean PCOS teenagers: A follow-up comparison between Myo-Inositol and oral contraceptives. Eur. Rev. Med. Pharm. Sci..

[86] Merviel P., James P., Bouée S., Le Guillou M., Rince C., Nachtergaele C., Kerlan V. (2021). Impact of myo-inositol treatment in women with polycystic ovary syndrome in assisted reproductive technologies. Reprod. Health.

[87] Elsaid S., Rubin-Kahana D.S., Kloiber S., Kennedy S.H., Chavez S., Le Foll B. (2022). Neurochemical Alterations in Social Anxiety Disorder (SAD): A Systematic Review of Proton Magnetic Resonance Spectroscopic Studies. Int. J. Mol. Sci..

[88] Taylor C.W., Tovey S.C. (2010). IP(3) receptors: Toward understanding their activation. Cold Spring Harb. Perspect. Biol..

[89] Cappelli V., Musacchio M.C., Bulfoni A., Morgante G., De Leo V. (2017). Natural molecules for the therapy of hyperandrogenism and metabolic disorders in PCOS. Eur. Rev. Med. Pharm. Sci..

[90] Benvenga S., Antonelli A. (2016). Inositol(s) in thyroid function, growth, and autoimmunity. Rev. Endocr. Metab. Disord..

[91] Pundir J., Psaroudakis D., Savnur P., Bhide P., Sabatini L., Teede H., Coomarasamy A., Thangaratinam S. (2018). Inositol treatment of anovulation in women with polycystic ovary syndrome: A meta-analysis of randomised trials. BJOG.

[92] Vartanyan E.V., Tsaturova K.A., Devyatova E.A., Mikhaylyukova A.S., Levin V.A., Petuhova N.L., Markin A.V., Steptsova E.M. (2017). Improvement in quality of oocytes in polycystic ovarian syndrome in programs of in vitro fertilization. Gynecol. Endocrinol..

[93] Regidor P.A., Schindler A.E. (2016). Myoinositol as a Safe and Alternative Approach in the Treatment of Infertile PCOS Women: A German Observational Study. Int. J. Endocrinol..

[94] Zacchè M.M., Caputo L., Filippis S., Zacchè G., Dindelli M., Ferrari A. (2009). Efficacy of myo-inositol in the treatment of cutaneous disorders in young women with polycystic ovary syndrome. Gynecol. Endocrinol..

[95] Minozzi M., D’Andrea G., Unfer V. (2008). Treatment of hirsutism with myo-inositol: A prospective clinical study. Reprod. Biomed. Online.

[96] Vitagliano A., Saccone G., Cosmi E., Visentin S., Dessole F., Ambrosini G., Berghella V. (2019). Inositol for the prevention of gestational diabetes: A systematic review and meta-analysis of randomized controlled trials. Arch. Gynecol. Obs..

[97] Carlomagno G., Unfer V. (2011). Inositol safety: Clinical evidences. Eur. Rev. Med. Pharm. Sci..

[98] Genazzani A.D., Santagni S., Ricchieri F., Campedelli A., Rattighieri E., Chierchia E., Marini G., Despini G., Prati A., Simoncini T. (2014). Myo-inositol modulates insulin and luteinizing hormone secretion in normal weight patients with polycystic ovary syndrome. J. Obs. Gynaecol. Res..

[99] Unfer V., Facchinetti F., Orrù B., Giordani B., Nestler J. (2017). Myo-inositol effects in women with PCOS: A meta-analysis of randomized controlled trials. Endocr. Connect..

[100] Zhang J.Q., Xing C., He B. (2022). Short period-administration of myo-inositol and metformin on hormonal and glycolipid profiles in patients with polycystic ovary syndrome: A systematic review and updated meta-analysis of randomized controlled trials. Eur. Rev. Med. Pharm. Sci..

[101] Unfer V., Porcaro G. (2014). Updates on the myo-inositol plus D-chiro-inositol combined therapy in polycystic ovary syndrome. Expert Rev. Clin. Pharm..

[102] Crouch E., Wright J.R. (2001). Surfactant proteins a and d and pulmonary host defense. Annu. Rev. Physiol..

[103] Lugogo N., Francisco D., Addison K.J., Manne A., Pederson W., Ingram J.L., Green C.L., Suratt B.T., Lee J.J., Sunday M.E. (2018). Obese asthmatic patients have decreased surfactant protein A levels: Mechanisms and implications. J. Allergy Clin. Immunol..

[104] Kwatia M.A., Doyle C.B., Cho W., Enhorning G., Ackerman S.J. (2007). Combined activities of secretory phospholipases and eosinophil lysophospholipases induce pulmonary surfactant dysfunction by phospholipid hydrolysis. J. Allergy Clin. Immunol..

[105] Ogasawara Y., Kuroki Y., Akino T. (1992). Pulmonary surfactant protein D specifically binds to phosphatidylinositol. J. Biol. Chem..

[106] Ogasawara Y., McCormack F.X., Mason R.J., Voelker D.R. (1994). Chimeras of surfactant proteins A and D identify the carbohydrate recognition domains as essential for phospholipid interaction. J. Biol. Chem..

[107] Lam S., McWilliams A., LeRiche J., MacAulay C., Wattenberg L., Szabo E. (2006). A phase I study of myo-inositol for lung cancer chemoprevention. Cancer Epidemiol. Biomark. Prev..

[108] Lam S., Mandrekar S.J., Gesthalter Y., Allen Ziegler K.L., Seisler D.K., Midthun D.E., Mao J.T., Aubry M.C., McWilliams A., Sin D.D. (2016). Cancer Prevention Network. A Randomized Phase IIb Trial of myo-Inositol in Smokers with Bronchial Dysplasia. Cancer Prev. Res..

[109] Laganà A.S., Unfer V., Garzon S., Bizzarri M. (2020). Role of inositol to improve surfactant functions and reduce IL-6 levels: A potential adjuvant strategy for SARS-CoV-2 pneumonia?. Med. Hypotheses.

[110] Hallman M., Bry K., Hoppu K., Lappi M., Pohjavuori M. (1992). Inositol supplementation in premature infants with respiratory distress syndrome. N. Engl. J. Med..

[111] Spengler D., Winoto-Morbach S., Kupsch S., Vock C., Blöchle K., Frank S., Rintz N., Diekötter M., Janga H., Weckmann M. (2018). Novel therapeutic roles for surfactant-inositols and-phosphatidylglycerols in a neonatal piglet ARDS model: A translational study. Am. J. Physiol. Lung Cell. Mol. Physiol..

[112] Howlett A., Ohlsson A., Plakkal N. (2015). Inositol in preterm infants at risk for or having respiratory distress syndrome. Cochrane Database Syst. Rev..

[113] Baldassarre M.P.A., Di Tomo P., Centorame G., Pandolfi A., Di Pietro N., Consoli A., Formoso G. (2021). Myoinositol Reduces Inflammation and Oxidative Stress in Human Endothelial Cells Exposed In Vivo to Chronic Hyperglycemia. Nutrients.

[114] Gagliardo R., Chanez P., Mathieu M., Bruno A., Costanzo G., Gougat C., Vachier I., Bousquet J., Bonsignore G., Vignola A.M. (2003). Persistent activation of nuclear factor-kappaB signaling pathway in severe uncontrolled asthma. Am. J. Respir. Crit. Care Med..

[115] Alipour S., Mahmoudi L., Ahmadi F. (2022). Pulmonary drug delivery: An effective and convenient delivery route to combat COVID-19. Drug Deliv. Transl. Res..

[116] Spiga A. (2021). Nebulized myo-Inositol increases oxygen saturation and relieves symptoms in patients with airways diseases. IJMDAT.

[117] Orrù B., Circo R., Logoteta P., Petousis S., Carlomagno G. (2017). Finding the best therapeutic approach for PCOS: The importance of inositol(s) bioavailability. Eur. Rev. Med. Pharm. Sci..

[118] Dinicola S., Minini M., Unfer V., Verna R., Cucina A., Bizzarri M. (2017). Nutritional and Acquired Deficiencies in Inositol Bioavailability. Correlations with Metabolic Disorders. Int. J. Mol. Sci..

[119] De Grazia S., Carlomagno G., Unfer V., Cavalli P. (2012). Myo-inositol soft gel capsules may prevent the risk of coffee-induced neural tube defects. Expert Opin. Drug Deliv..

[120] Kamenov Z., Kolarov G., Gateva A., Carlomagno G., Genazzani A.D. (2015). Ovulation induction with myo-inositol alone and in combination with clomiphene citrate in polycystic ovarian syndrome patients with insulin resistance. Gynecol. Endocrinol..

[121] Raffone E., Rizzo P., Benedetto V. (2010). Insulin sensitiser agents alone and in co-treatment with r-FSH for ovulation induction in PCOS women. Gynecol. Endocrinol..

[122] Gerli S., Papaleo E., Ferrari A., Di Renzo G.C. (2007). Randomized, double blind placebo-controlled trial: Effects of myo-inositol on ovarian function and metabolic factors in women with PCOS. Eur. Rev. Med. Pharm. Sci..

[123] Grases F., Simonet B.M., Vucenik I., Prieto R.M., Costa-Bauzá A., March J.G., Shamsuddin A.M. (2001). Absorption and excretion of orally administered inositol hexaphosphate (IP(6) or phytate) in humans. Biofactors.

[124] Yamaguchi M., Yoshida K., Uchida M. (2009). Novel functions of bovine milk-derived alpha-lactalbumin: Anti-nociceptive and anti-inflammatory activity caused by inhibiting cyclooxygenase-2 and phospholipase A2. Biol. Pharm. Bull..

[125] Monastra G., Sambuy Y., Ferruzza S., Ferrari D., Ranaldi G. (2018). Alpha-lactalbumin Effect on Myo-inositol Intestinal Absorption: In vivo and In vitro. Curr. Drug Deliv..

[126] Montanino Oliva M., Buonomo G., Calcagno M., Unfer V. (2018). Effects of myo-inositol plus alpha-lactalbumin in myo-inositol-resistant PCOS women. J. Ovarian Res..

[127] Manish R.P., Jignesh S., Varsha N. (2022). The Role of Alpha-Lactalbumin with Myoinositol in the Treatment of PCOS: A Review. Asian Res. J. Gynaecol. Obs..

[128] Izumi H., Ishizuka S., Inafune A., Hira T., Ozawa K., Shimizu T., Takase M., Hara H. (2009). Alpha-Lactalbumin hydrolysate stimulates glucagon-like peptide-2 secretion and small intestinal growth in suckling rats. J. Nutr..

[129] Förster C. (2008). Tight junctions and the modulation of barrier function in disease. Histochem. Cell Biol..

[130] Lemmer H.J., Hamman J.H. (2013). Paracellular drug absorption enhancement through tight junction modulation. Expert Opin. Drug Deliv..

[131] Cardinale V., Lepore E., Basciani S., Artale S., Nordio M., Bizzarri M., Unfer V. (2022). Positive Effects of α-Lactalbumin in the Management of Symptoms of Polycystic Ovary Syndrome. Nutrients.

[132] D’Anna R., Corrado F., Loddo S., Gullo G., Giunta L., Di Benedetto A. (2021). Myoinositol plus α-lactalbumin supplementation, insulin resistance and birth outcomes in women with gestational diabetes mellitus: A randomized, controlled study. Sci. Rep..

[133] Hernandez Marin I., Picconi O., Laganà A.S., Costabile L., Unfer V. (2021). A multicenter clinical study with myo-inositol and alpha-lactalbumin in Mexican and Italian PCOS patients. Eur. Rev. Med. Pharm. Sci..

